# Adsorption of crystal violet dye from synthetic wastewater by ball-milled royal palm leaf sheath

**DOI:** 10.1038/s41598-024-52395-8

**Published:** 2024-03-04

**Authors:** Neloy Sen, Nawrin Rahman Shefa, Kismot Reza, Sk Md Ali Zaker Shawon, Md. Wasikur Rahman

**Affiliations:** 1 Department of Chemical Engineering, Jashore University of Science and Technology, Jashore, 7408 Bangladesh; 2 Department of Chemical and Biomolecular Engineering, Vanderbilt University, Nashville, TN 37235 USA; 3 Department of Physics and Astronomy, University of Texas Rio Grande Valley, 1201 West University Drive, Edinburg, TX 78539 USA

**Keywords:** Chemistry, Materials science

## Abstract

The current study shows that using a batch approach to remove crystal violet dye from synthetic wastewater is feasible when using royal palm leaf sheath powder as an adsorbent. In order to investigate the effects of many parameters, including starting concentration, pH effect, dye concentration, adsorbent dose, contact time, and temperature, experiments were carried out under various operating conditions. Maximum removal was obtained at pH 6 and at a concentration of 100 ppm, which are considered as ideal values. The influence of pH and dye concentration was shown to be substantial. Langmuir, Freundlich, and Temkin isotherm models were fitted to equilibrium data. The Langmuir isotherm model, which showed a maximum monolayer adsorption capacity of 454.5455 mg/g, best described the equilibrium data. The Pseudo-second-order kinetic model was found to closely resemble rather than the first-order and intra-particle diffusion models. Standard enthalpy (∆H◦**)**, entropy (**∆S◦**), and free energy (∆G◦) were evaluated as thermodynamic parameters. It was discovered that the adsorption contact was endothermic in nature. The outcomes highlight the applicability of the inexpensive, locally accessible adsorbent in the specialty area of wastewater treatment and can be used in commercial dye-enriched effluent.

## Introduction

Humans have benefited from rapid industrialization, and evolution in nanotechnology but it has also negatively harmed the environment. Numerous sectors, particularly the dye industry produce huge amounts of wastewater containing harmful chemicals, and nanomaterials which are to blame for the sharp increase in pollution. A new strategy is evolving, with the focus turning to resource recovery from such wastewater and its management in a sustainable manner, notwithstanding the enormous advances made in the treatment and organization of such wastewater through chemical or biological processes. Adsorption, advanced oxidation, membrane filtration, microbial technology, electrochemical and photocatalytic degradation, etc. are some of the most cutting-edge technical and scientific breakthroughs for treating dye industry effluent. Microbial degradation stands out among these technologies as being particularly promising for resource recovery and sustainability^1^. The production of toxic sludge and high operational and maintenance expenses are two drawbacks of most of these techniques. So, to remove dyes from effluents, technological developments are used^2^. Adsorption offers advantages over other technologies due to its straightforward design and potential for a modest initial investment as well as minimal acreage requirements. Adsorption process draws significant attention and frequently used to clean up organic and inorganic impurities from industrial wastewater^3^. Bio-adsorbents are coming under green chemistry. It’s economical, environmentally friendly, and non-threatening to nature. The bio-adsorbents procedure is equivalent to other physicochemical treatment methods, convenient to use, and extremely effective while producing less sludge^4^.

A triphenylmethane dye called crystal violet (CV) has been widely utilized as a biological stain in human and veterinary medicine, as a textile dye in the textile processing industry, and to give paints and printing ink a rich violet hue. Additionally, CV is utilized in medical solutions as a mutagenic and bacteriostatic agent as well as an antibacterial agent to stop the growth of fungi in chicken feed. Despite vast applications, CV has been described as a stubborn dye molecule that has harmful effects and lasts for long time in the environment. It plays a role in selective fish species as a mitotic toxin, carcinogen, and clastogene that encourage the growth of tumors. CV is, therefore, considered a bio-hazardous chemical. Although there are a number of physicochemical methods to remove CV, including adsorption, coagulation, and ion-pair extraction which still have drawbacks for the comprehensive removal of CV from industrial wastewater in addition to produce significant amount of sludge containing secondary pollutants^5^. Nevertheless, biological techniques are thought of as economical and environmentally benign for industrial wastewater treatment, but these techniques also have some limits. Therefore, the creation of such eco-friendly and affordable biological treatment techniques that can successfully remove the dye from industrial wastewater is urgently required to protect the environment and the health of people and animals^6^. Removal of CV by adsorption here comes to rescue.

The decorative plant species commonly known as the Cuban royal palm or Florida royal palm, or simply royal palm (*Roystonea regia*) is often used in urban environments. The palms naturally die at the end of their life cycle, becoming waste as well as leftovers from the timber industry. The Royal Palm Leaf Sheath (RPLS) averages 161.60 cm in length, 53.47 cm in breadth, and 3.54 mm in thickness, with a thickness gradient from the core to the margin. Vascular bundles and parenchyma are the two main parts of the leaf sheath^7^. Involved in photosynthesis, storage, or transport, parenchyma is a crucial component of vascular tissue^8^. The cellulose content and crystallinity index of the RPLS fiber is around 56% which can be compared to naturally available lignocellulosic fibers; however, lower in density, a potential source of lightweight composite materials. Considering the overall fiber properties of the royal palm, RPLS is a useful material for textile, polymer composites, pulp and paper industries as a prospective cost-effective adsorbent for wastewater treatment.

Ball milling is a state-of-the-art surface grinding method that grinds materials, for example RPLS, into extremely fine powders. During milling, localized high pressure is generated through the collision among the tiny rigid balls in a concealed container. Several factors are accountable for the quality of dispersion, e.g. milling time, rotational speed, dimension of balls, and balls to solid weight ratio. Upon considering all the conditions, solid materials can be ground to only 100 nm particle sizes. Therefore, ball milling is an economically feasible approach as well as widely employed in industry, especially in nanocomposite production sectors. Milling is a mechanical process with low energy consumption, and increases specific surface area and oxygen-containing functional groups on the surfaces of the particles^9^.

In fact, huge work has been reported in the literature on the removal of toxic chemicals by naturally available low-priced adsorbents from industrial wastewater^5,10–12^; however, the adsorption of recalcitrant CV dye using RPLS powder has not been reported in details so far. Besides, the unique novelty of this study is microstructuring of the adsorbent by ball milling and comparing the adsorption parameters between RPLS and BMRPLS with the synthetic dye towards practical viewpoint.

In the current study, the main objective is to develop a sustainable cost–effective adsorbent from locally obtainable royal palm to discharge CV from industrial wastewater. Various experimental parameters were investigated along with adsorption isotherms, kinetics, diffusion, and thermodynamics of the process to understand the real pertinence of the as prepared RPLS adsorbent.

## Materials and methods

### Chemicals

The full set of chemicals was of the analytical reagent grade. By dissolving in distilled water, stock solutions of the Crystal Violet dye were prepared. Accurately weighed dyes were dissolved in distilled water to make stock solution of 500 ppm dye concentration. The dye stock solution was accurately diluted to the required initial concentrations to produce the experimental solutions. Original pH of each dye solution was adjusted using 0.1 M HCl and 0.1 M NaOH.

### Adsorbent preparation and characterization

**Figure 1:**
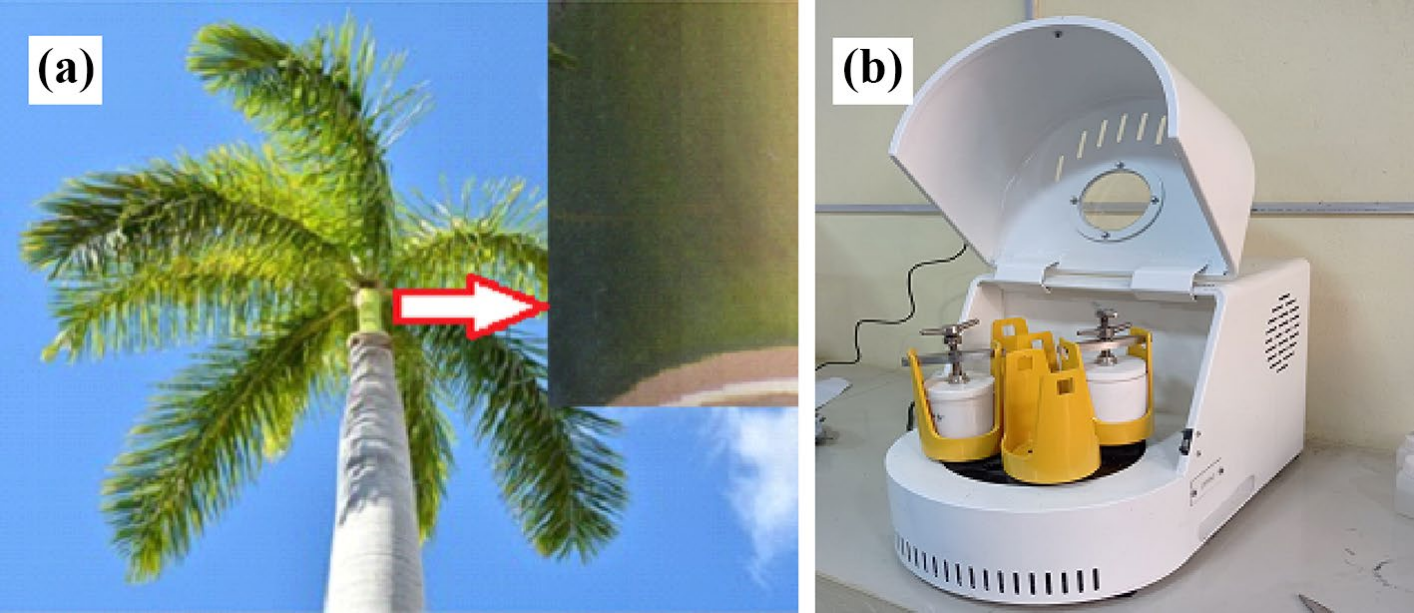
(**a**) Royal palm leaf sheath (**b**) Planetary ball mill.

The natural RPLS (Fig. 1a) was obtained from a campus garden in Jashore University of Science and Technology, Bangladesh and washed with distilled water. Then the leaf sheath dried at 70 °C in a hot air oven for 4 h. The dried leaf sheath was grounded by a grinder and sieved to obtain particles of desired mesh size. Then RPLS powder was kept in a hot air oven to remove moisture overnight at 60 °C. The as prepared RPLS was subjected to feed into a planetary ball mill (DECO-PBM-V-0.4L, Dimension 502 × 300 × 296 mm^3^) (Fig. 1b) with PTFE high-pressure milling vial and SS balls. It was made sure that the ball mill was perfectly sealed. Later, the ball mill was run for 5 h at an operating speed of 800 rpm at room temperature with powder-ball ratio 1:10. Then ball milled RPLS (BMRPLS) powder was stored into an airtight box for further use.

RPLS was analyzed by Fourier Transform Infrared (FTIR) Spectroscopy (Model: FTIR 2000, Shimadzu, Japan) and spectra were recorded between 400 and 4000 cm^–1^. KBr disks having 150 mg around 2% of samples were prepared. Scanning electron microscopy (SEM) of the adsorbent was carried out to reveal the morphology of the adsorbent. SEM was obtained by putting sample on Leo 435 VP.

### Point of zero charge (pHpzc) determination

Salt addition procedure was achieved by adding substrate of identical amounts to a series of solutions at different pH values. In a series of 250 mL conical flasks, 200 mg of the test sample was added to 50 mL of 0.1 M KNO_3_ solution. pH was adjusted with 0.1 M HCl and 0.1 M NaOH as required to get hold of the appropriate pH range of 3, 5, 7, 9, 10. pH of the solutions in each flask were denoted as pHi. Test samples were shaken at 200 rpm for 2 h using an orbital shaker. Once settled down, pH of the solutions in each flask were estimated and represented as pH_f_. Then a plot of ΔpH (pH_f_–pH_i_) against pH_i_ was carried out to obtain the point of zero charge (pHpzc).

### pH of dye solution

To observe the effect of pH on the adsorption process, various pH dye solutions were selected such as pH 2, 3, 4, 5, 6, 7, 8, 9, 10. CV dye solution with a pH of approximately 6 was maintained with distilled water. 100 mL 0.1 M HCl and equal volume 0.1 M NaOH were used to decrease or increase the pH of the solution to achieve the desired pH. Then 40 mg of BMRPLS adsorbent was added into each 25 mL 100 ppm CV dye solutions of different pH values followed by constant shaking at 200 rpm for 1 h. After that the solutions were centrifuged at 400 rpm for 5 min in view of determining the optimum pH of the test samples for maximum dye removal.

### Batch adsorption studies

In a 100 mL conical flask, 40 mg of biosorbent was mixed with 25 mL of dye solution (100 ppm) for each experiment. The flasks were agitated at 200 rpm for several spans of time at 25 °C e.g. 10, 20, 30, 40, 50, 60, 70 min. Following the treatment, biosorbents were separated by centrifuging at 4000 rpm. In view of 200 ppm dye solutions, experiments were done to determine the impact of pH on biosorption at pH 2–10. Utilizing various concentrations, the impact of the initial dye concentrations (50, 100, 150, 200, 250 ppm) was studied. After the treatment, the residual dye concentrations in the solutions were measured using UV–vis spectroscopy (HACH–DR–4000). The optimum wavelength (λ_max_) for CV dye was 591 nm.

### Data processing and analysis

The effectiveness of each biosorbent in adsorbing the dye from the medium was measured by the removal percentage of the various biosorbents. The following equation (Eq. 1) was used to express adsorption efficiency:

1 $$\% {\text{ Removal of CV }} = \times { 1}00\% ,$$

where C_0_ and C_e_ are the initial and equilibrium concentrations (mg/L) of CV, respectively. The amount of adsorption at equilibrium, q_e_ (mg/g), calculated by following equation (Eq. 2):

2 $${q}_{e}=\frac{\left({C}_{0}-{C}_{e}\right)V}{m},$$

where V and m correspond to the volume (L) of the solution and mass (g) of dry absorbent used^13^.

### Adsorption isotherms

Adsorption isotherms relate solute concentration on an adsorbent surface to its equilibrium concentration in the liquid in which it is in contact. The most suitable correlation for the equilibrium curves must be established for eliminating CV from aqueous solution^14,15^. Three significant isotherms were used in this work to fit the equilibrium data such as Langmuir, Freundlich and Temkin isotherms, which are mentioned in Table 1.

**Table 1:** Different adsorption isotherm model equations that fit the equilibrium data.

Model	Equations	Plot	Notations	Reference
Langmuir isotherm	$\frac{1}{{q}_{e}}=\frac{1}{{q}_{max}}+\frac{1}{{K}_{L}{q}_{max}{C}_{e}}$ ${R}_{L}=\frac{1}{1+(1+{K}_{L}{C}_{0})}$	$\frac{1}{{q}_{e}} vs \frac{1}{C{}_{e}}$	c_e_ = the equilibrium concentration of adsorbate (mg/L) q_e_ = the amount of metal adsorbed per gram of adsorbent at equilibrium (mg/g) q_max_ = maximum monolayer coverage capacity (mg/g) K_L_ = Langmuir isotherm constant (L/mg) R_L_ = Separation factor	^16^
Freundlich isotherm	$log{q}_{e}=log{K}_{f}+\frac{1}{n}log{C}_{e}$	$log{q}_{e} vs log{C}_{e}$	K_f_ = Freundlich isotherm constant (mg/g) n = adsorption intensity c_e_ = the equilibrium concentration of adsorbate (mg/L) q_e_ = the amount of metal adsorbed per gram of adsorbent at equilibrium (mg/g)	^17^
Temkin isotherm	${q}_{e}=B lnA+\mathit{Bln}{C}_{e}$ $B=\frac{RT}{b}$	${q}_{e}$ vs $\mathit{ln}{C}_{e}$	A = Temkin constant associated with maximum binding energy (L/g) B = Temkin constant associated with adsorption heat (J/mol) R = Universal gas constant (8.314 J/mol) T = Absolute temperature (K) b = Temkin constant related to the heat of adsorption (J/mol)	^18^

### Adsorption kinetics

To describe the dynamic adsorption interactions between the adsorbent and dye, kinetic model analysis is used^19,20^. The following Table 2 contains intraparticle, first-order and second-order kinetic models, and experimental data were employed to analyze the kinetics of CV dye adsorption.

**Table 2:** Adsorption kinetic model equations.

Model	Equation	Parameter	Reference
Pseudo-first-order	${\text{log}}({q}_{e}-{q}_{t})={\text{log}}{q}_{e}-\frac{{k}_{1}}{2.303}t$	q_e_ = the amount of adsorbate adsorbed on adsorbent at equilibrium (mg/g) q_t_ = the amount of adsorbate adsorbed on adsorbent at time (mg/g) k_1_ = the pseudo-first-order rate constant (min^–1^) of adsorption	^21^
Pseudo-second-order	$\frac{t}{{q}_{t}}=\frac{1}{{k}_{2}{q}_{e}^{2}}+\frac{t}{{q}_{e}}$	K_2_ = the pseudo-second-order rate constant (min g/mg) q_e_ = the amount of dye adsorbed (mg/g) at equilibrium q_t_ = the amount of dye adsorbed (mg/g) at time t	^22^
Intraparticle diffusion	${q}_{t}={k}_{i}{t}^{0.5}$+ C	Q_t_ = the adsorption capacity at any time (mg/g) K_in_ = the intra-particle diffusion rate constant (mg/gmin^1/2^) C = constant for the boundary layer effect	^23^

### Adsorption thermodynamics

To assess the viability and exothermic characteristics of the adsorption process, the thermodynamic parameters e.g. change in standard free energy (∆G°), enthalpy (∆H°), and entropy (∆S°) were evaluated. The following equations (Eqs. 3 and 4) link the change in Gibb’s free energy to the equilibrium constant of the process^24^:

3 $$\Delta {\text{G}}^\circ \, = \, - {\text{RT ln K}}_{{\text{d}}} .$$

According to thermodynamic and Gibb’s free energy change,

4 $$ln{k}_{d}=\frac{\Delta {\text{S}}^\circ }{R}+\frac{\Delta {\text{H}}^\circ }{RT},$$

where T (K) and R (J/mol.K) correspond to the absolute solution temperature and universal gas constant, and k_d_ is the distribution co-efficient which may be computed as (Eq. 5):

5 $${k}_{d}=\frac{{C}_{Ae}}{{C}_{e}},$$

where ${C}_{Ae}$ is the amount of CV absorbed at equilibrium (mg/L), ${C}_{e}$ is the concentration of CV remaining in the solution at equilibrium, ∆G° can be calculated from Ref.^25^.

### Regeneration

The regeneration experiments were carried out in 100 mL Erlenmeyer flasks. The RPLS and BMRPLS samples with adsorbed CV were centrifuged, washed, dried, and regenerated with DW consecutively for 1 h at 25 °C. The regenerated RPLS and BMRPLS were then poured back into the flask for adsorption, with the adsorption-regeneration process repeated three times. This data was used to calculate the CV uptake rate.

### Ethical approval

The collection and use of Royal palm plant materials in this study is carried out in accordance with Institutional guidelines and regulations.

## Results and discussion

### Characterization of adsorbent

#### FTIR of RPLS adsorbent

**Figure 2:**
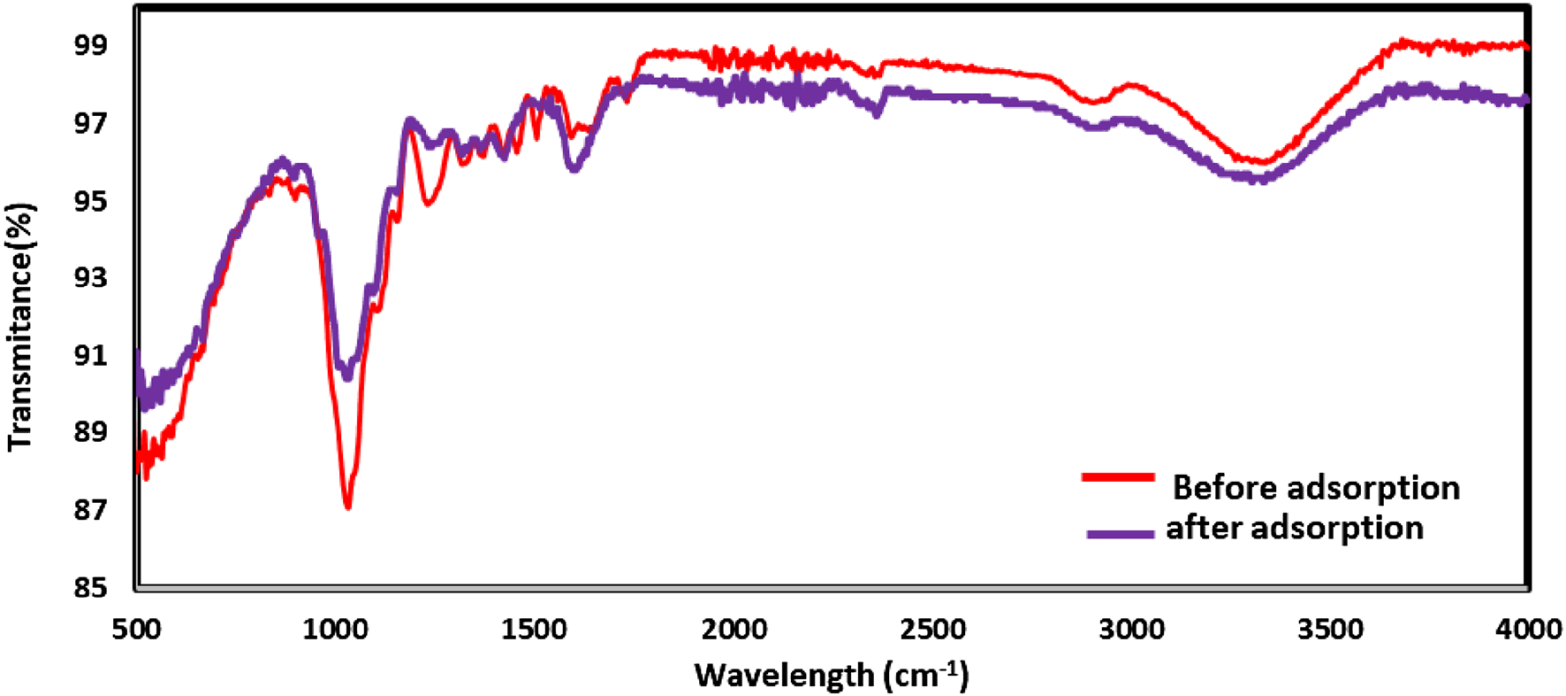
FTIR spectra of BMRPLS before and after adsorption of CV.

A popular technique for identifying the functional groups that act as adsorption sites is FTIR spectroscopy. The FTIR spectra of the adsorbent before and after CV adsorption are displayed in Fig. 2. The complexity of the material under study is shown by the analysis of the FTIR spectrum, which reveals the presence of several peaks in the wavelength range from 4000 to 500 cm^–1^. Before adsorption, the broad transmittance band at approximately 3337 cm^–1^ and 2916 cm^–1^ correspond to the elongation of the O–H groups and C–H groups. The band at 1732 cm^–1^ relates the elongation vibration of the nonconjugated C=O bonds and that at 1604 cm^–1^ is characteristic for the elongation of the C=C bonds of aromatic compounds, a characteristic that is mainly caused by building block of the leaf sheath. FTIR spectra before and after CR adsorption on the biomass show some distinctive properties. The infrared band intensities fluctuate noticeably in the spectrum that results from the adsorption of CV, despite no change in band locations. The distinctive peaks observed from 1600 to 1100 cm^–1^ differentiate interaction between CV dye and BMRPLS. The FTIR spectra modification and the findings point out that the process at hand was a form of CV inclusion with the functional groups of the adsorbent present. In fact, change in intensity of the peaks observed before and after adsorption is a function of quantitative extent^5^.

#### SEM of RPLS adsorbent

**Figure 3:**
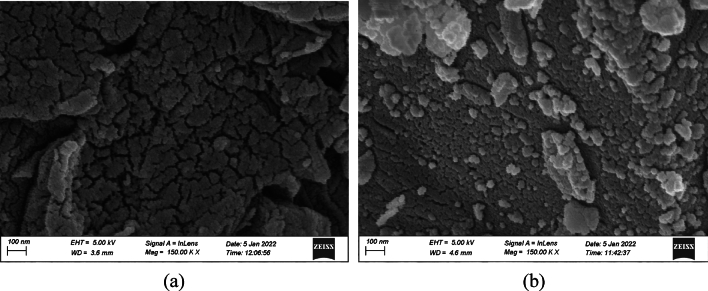
SEM images of BMRPLS (**a**) before and (**b**) after adsorption.

SEM was carried out to examine the surface morphology of BMRPLS before and after CV dye adsorption as shown in Fig. 3a,b with 150 K magnification at 5 kV. It can be revealed from the images that BMRPLS having huge pores and cracks on the surface (Fig. 3a) facilitate adsorption process^12,26^. Moreover, the irregular surface structure has been saturated by the dye molecules after adsorption as clearly evidenced in Fig. 3b. This phenomenon was also reported elsewhere^27^.

##### Point of zero charge

**Figure 4:**
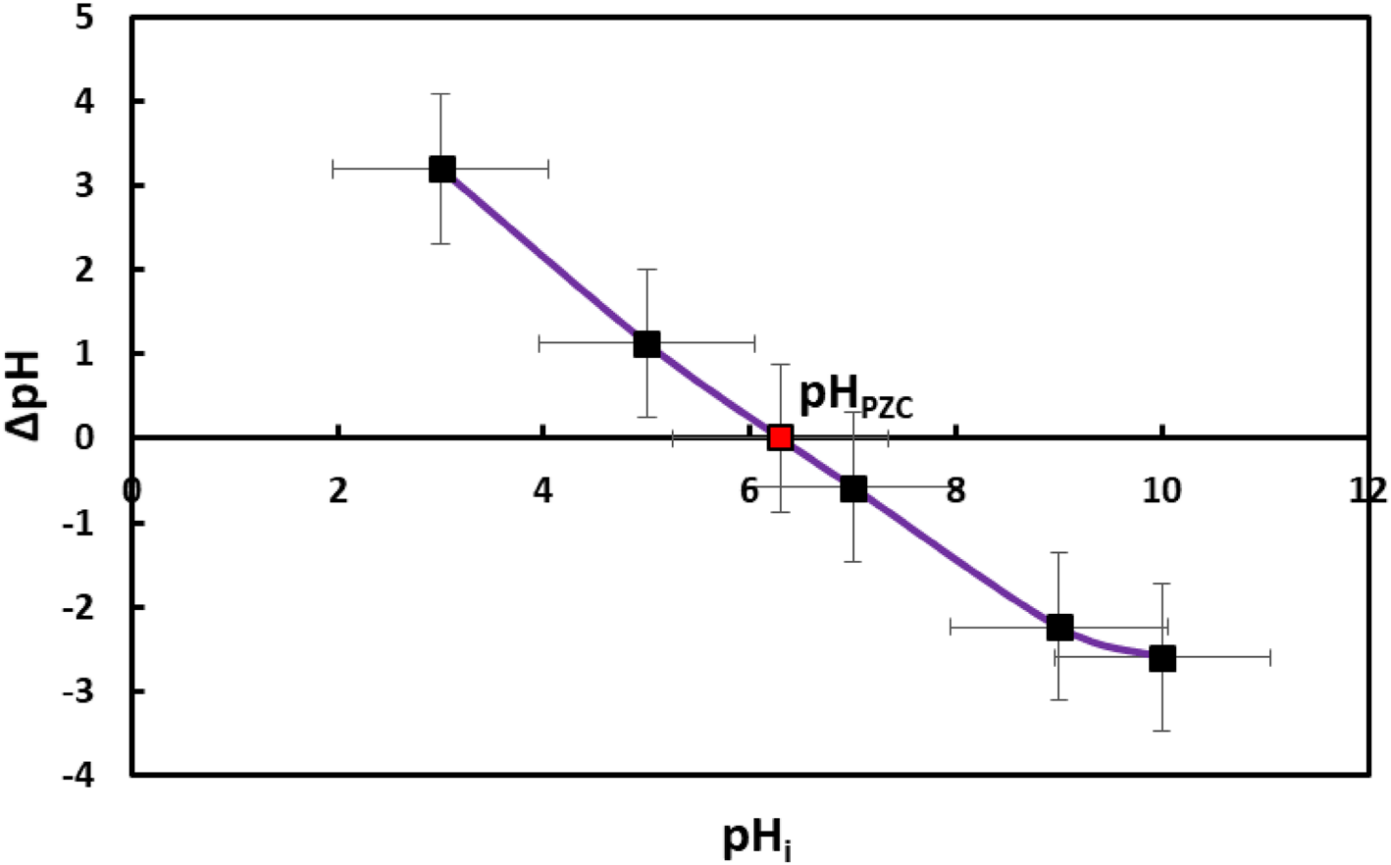
Point of zero charge (pH_PZC_).

Figure 4 displays the experimental curves with standard deviation obtained by using the salt addition approach. The pHpzc of the suspended solid is calculated as the result of intersecting the starting pH (pHi, x-axis) with the ΔpH = 0 line (y-axis). The pH and conditions of the medium at which the adsorbent surface charge density is zero are known as the point of zero charge (pHpzc). The pHpzc in the current investigation was found 6.3. As a result, the adsorbent surface has a primarily positive charge (cationic) when the solution pH is lower than pHpzc = 6.3; whereas, the net surface charge is negative (anionic) when the pH is higher than 6.3 (pHpzc > 6.3)^28^.

### Effect of solution pH

**Figure 5:**
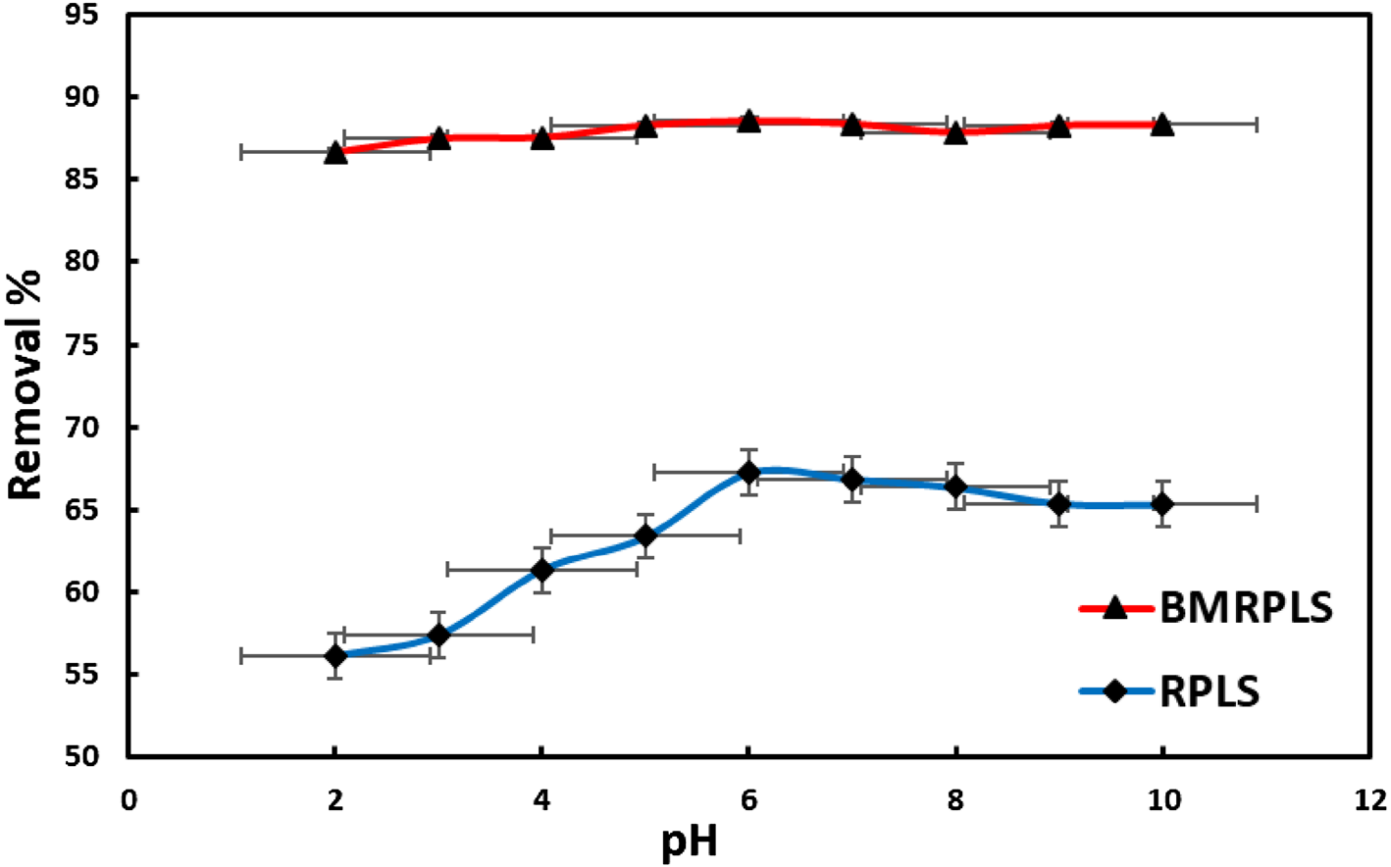
Effect of solution pH on the percentage removal of CV.

Since the pH of the adsorbate-adsorbent system plays such a critical role in the entire adsorption process, several researchers investigated the variations in dye adsorption on adsorbent over a wide range of pH values. Functional groups that can be protonated or deprotonated to produce distinct surface charges in various pH solutions may be present in both the adsorbent and the adsorbate, leading to electrostatic attraction or repulsion between the charged dyes and solid materials. Using RPLS before and after the separation process, we looked into how pH affected the removal of cationic dye from an aqueous solution^29^. By maintaining a fixed contact period of 60 min, ambient temperature of 25 °C, and adsorbent dose of 40 mg/L, the effect of starting pH on dye removal was examined at various initial CV concentrations. The impact of pH on BMRPLS ability to absorb dye is presented in Fig. 5 with standard deviation.

When the pH was raised from 2 to 6, it was found that the removal percentage increased substantially. Both are the series of RPLS that originated before and after ball milling. As pH increased, dye removal increased until it reached an optimal level of pH 6. CV removals were achieved 67.3% and 88.6% for RPLS and BMRPLS, respectively at the optimum value then adsorption marginally decreased. This trend is explained by the increase in negative charge density on the adsorbent in solutions with an acidic pH. These draw the positively charged CV dye molecule and RPLS together (before and after milled). The interaction between the positively charged dye molecules and the adsorbent decreases as pH rises because more negatively charged surfaces are available. Similar phenomenon is also discussed in elsewhere^5,11^. At higher pH values, the preponderance of OH^−^ ions in the solution generates a competition between the carbon surface and the solution OH^−^ ions for the CV cation which results in a decrease in the adsorption^30^.

### Effect of adsorbent dosage

**Figure 6:**
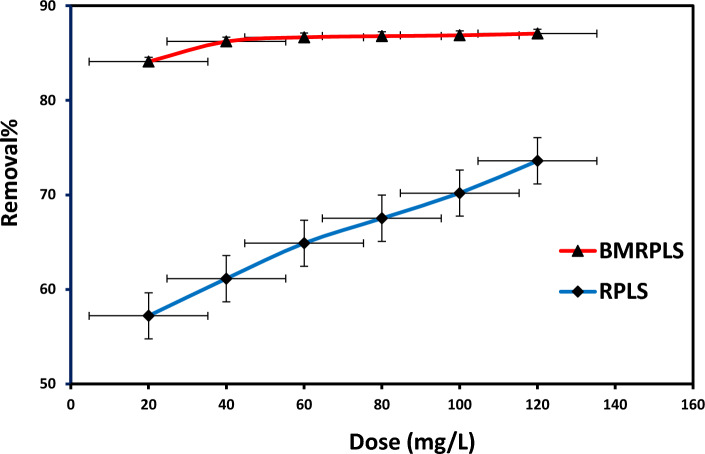
Effect of adsorbent dosage.

The capacity of the adsorbent for a given initial concentration of dye solutions was determined by the adsorbent dosage, which is a crucial parameter in adsorption experiments. By contacting 25 mL of dye solution with an initial dye concentration of 100 ppm for the adsorbent, for a contact time of 60 min at a temperature of 25 °C, a shaking speed of 200 rpm, and an optimal pH of 6, the effect of before and after BMRPLS dosages on the amount of dye adsorbed were investigated. Adsorbents were introduced in varying amounts (20, 40, 60, 80, 100, and 120 mg). The samples were centrifuged at 4000 rpm for 5 min after reaching equilibrium, and then they were given time to settle. The supernatant solutions were then gathered and examined using a UV–visible spectrophotometer. The impact of adsorbent doses on the CV dye removal by RPSLP is depicted in Fig. 6 with standard deviation.

With an increase in adsorbent doses, both before and after ball milling of RPLS powder, the percentage of dye removal increased. For instance, when the dosage was increased from 20 to 120 mg in RPLS with ball milling, an increase in CV removal from 84 to 87% was observed. At the parallel conditions, RPLS demonstrated dye removal lower (57 to 92%) than that of BMRPLS. Adsorption capacity achieved 417.9 and 353.2 mg/g for 120 mg adsorbent dose of BMRPLS and RPLS, respectively. The increase in adsorption with the addition of more adsorbent can be attributed to the availability of more adsorption sites and an increase in adsorption surface^31^. Prior to the implementation of BMRPLS, an increase in adsorbent mass from 60 to 80 mg revealed a marginally improved adsorption yield. This could be explained by the limited biomaterial aggregation, which ultimately results in a less effective surface area for adsorption.

### Effect of initial dye concentration

**Figure 7:**
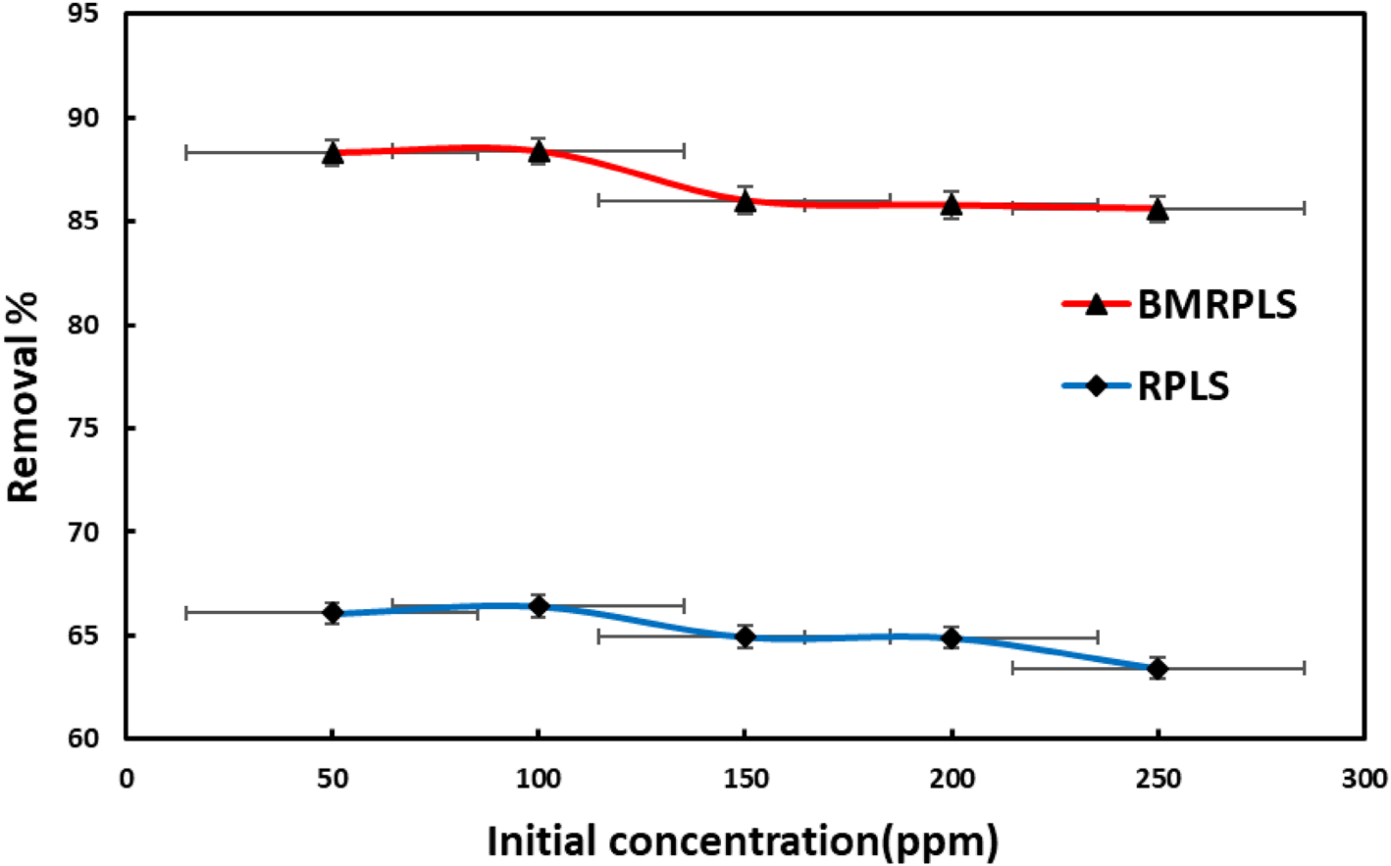
Effect of initial dye concentration.

The effect of initial concentration of CV on the adsorption of RPLS and BMRPLS is shown in Fig. 7 with standard deviation. The Figure shows that, both before and after ball milling, the removal percentage of CV drops as the starting concentration increases. The removal percentage falls off very gradually when the initial concentration of CV is low.

This outcome is attributable to the low concentration still falling within RPLS and BMRPLS adsorption range. The removal percentage visibly decreases if the initial CV concentration reaches a certain level. This is because RPLS and BMRPLS have relatively low adsorption capabilities. The adsorption site slowly fills up as the starting CV concentration gradually rises. The clearance percentage for RPLS and BMRPLS; however, can still be above 85% and 63%, respectively, when the starting concentration is 250 ppm. In the range from 50 to 250 ppm initial concentrations adsorption capacity increased from 20.6 to 99.1 mg/g for RPLS and 27.6 to 133.8 mg/g for BMRPLS. From it demonstrates that wastewater containing various amounts of CV may be effectively purified using RPLS and BMRPLS. However, BMRPLS has a higher removal rate to CV than RPLS, indicating that CV has a greater capacity for adsorption by BMRPLS^5^.

### Effect of contact time

**Figure 8:**
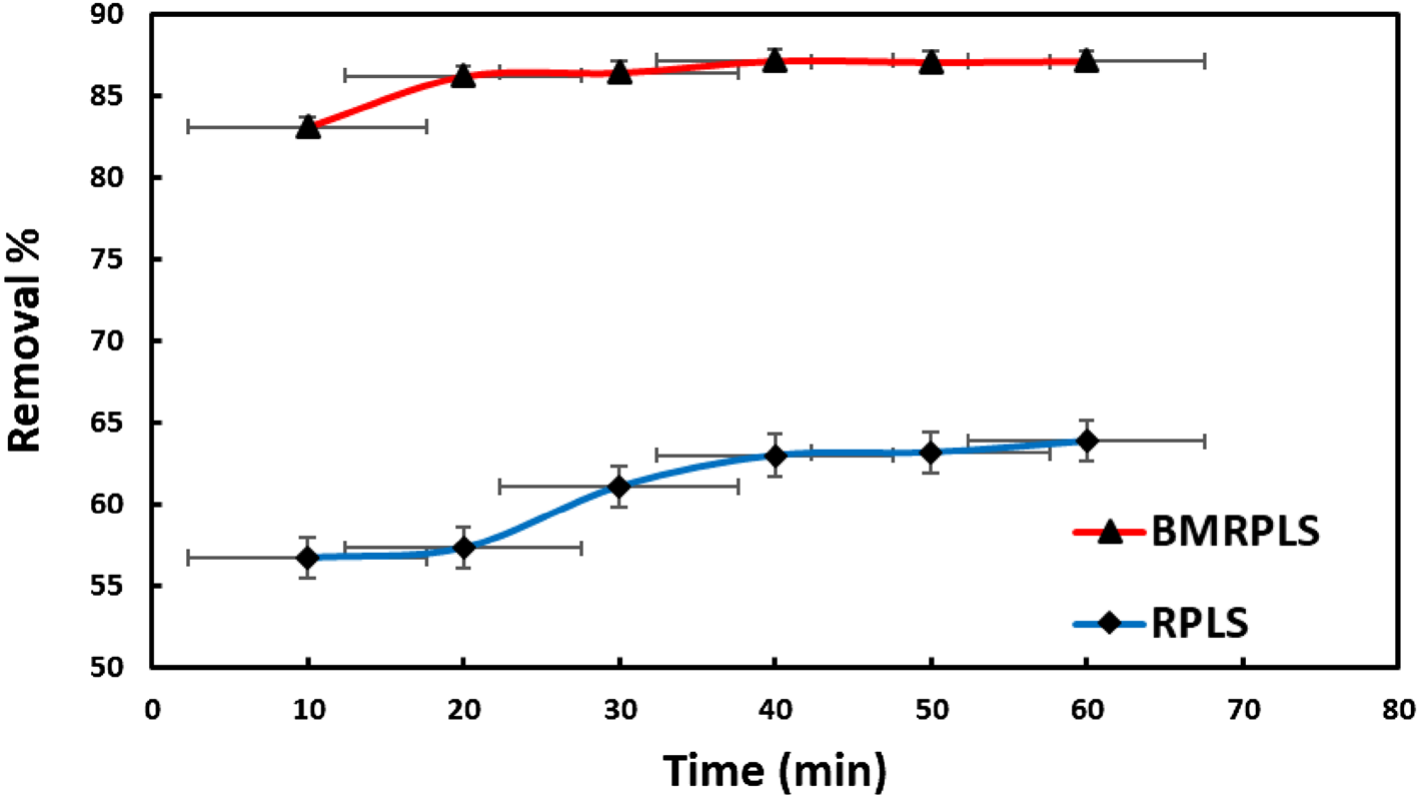
Effect of contact time on the percentage removal of CV.

The effect of contact time on the uptake of CV onto RPLS and BMRPLS was studied and is shown in Fig. 8 with standard deviation.

In the first 40 min, the rate of CV adsorption increased significantly, as can be shown, and the growth tended to be moderate after 60 min. For RPLS and BMRPLS, the maximum adsorption percentages were 87.2% and 63.9%, respectively. The unoccupied adsorption sites on the adsorbent surface may be responsible for these phenomena. There are a lot of empty surface sites accessible for adsorption in the early stages of sorption. Due to the repelling interactions between the solute particles on the solid surface and the bulk phase, the left over unfilled surface sites may be difficult to fill after some time has passed^32^.

### Effect of temperature

**Figure 9:**
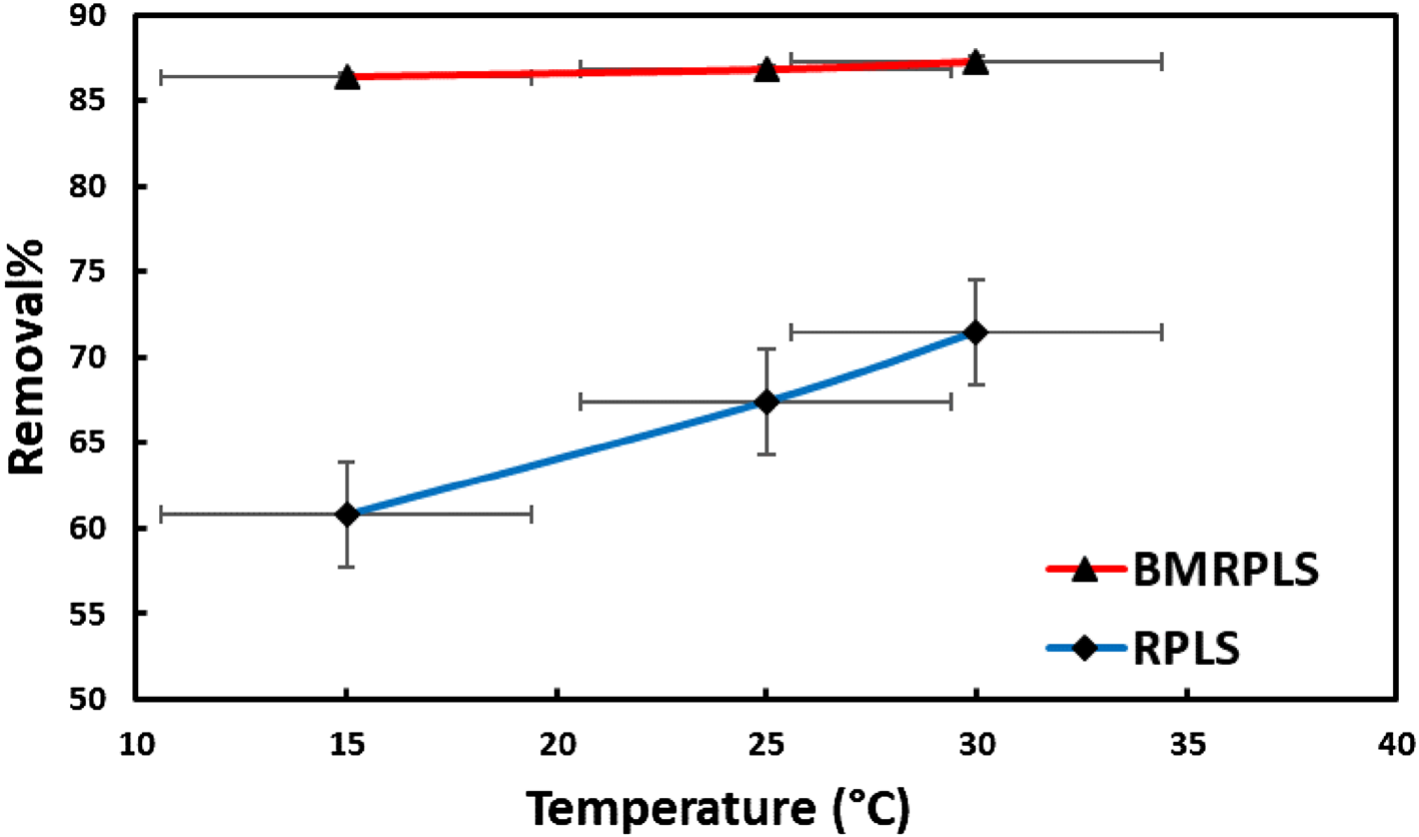
Effect of temperature on adsorption process.

Due to its impact on the surface characteristics of the adsorbent and the mobility and solubility of dye molecules in aqueous solutions, the temperature is a significant factor in the adsorption process. Additionally, as the temperature rises, the rate of adsorbate molecule diffusion through the external boundary layer of adsorbent particles and inside of their interior pores increases, and the viscosity of the solution decreases^33^. Effect of temperature on the adsorption of CV dye was investigated and the result was displayed in Fig. 9 with standard deviation.

The adsorption of CV by RPLS is temperature-dependent, as shown in Fig. 9. The adsorption mechanism was discovered to be temperature dependent, with the maximum CV absorption occurring at 30 °C. The enhanced ability of CV to adsorb RPLS as the solution temperature increased suggests that the process is endothermic. An increase in temperature improved the dispersion resistance of adsorbates to adsorbents by speeding up the advancement of the CV from the solution onto the vacant sites of RPLS and BMRPLS and decreasing the thickness of the surface layer of the leaf sheath.

### Adsorption isotherm

#### Langmuir adsorption isotherm

**Figure 10:**
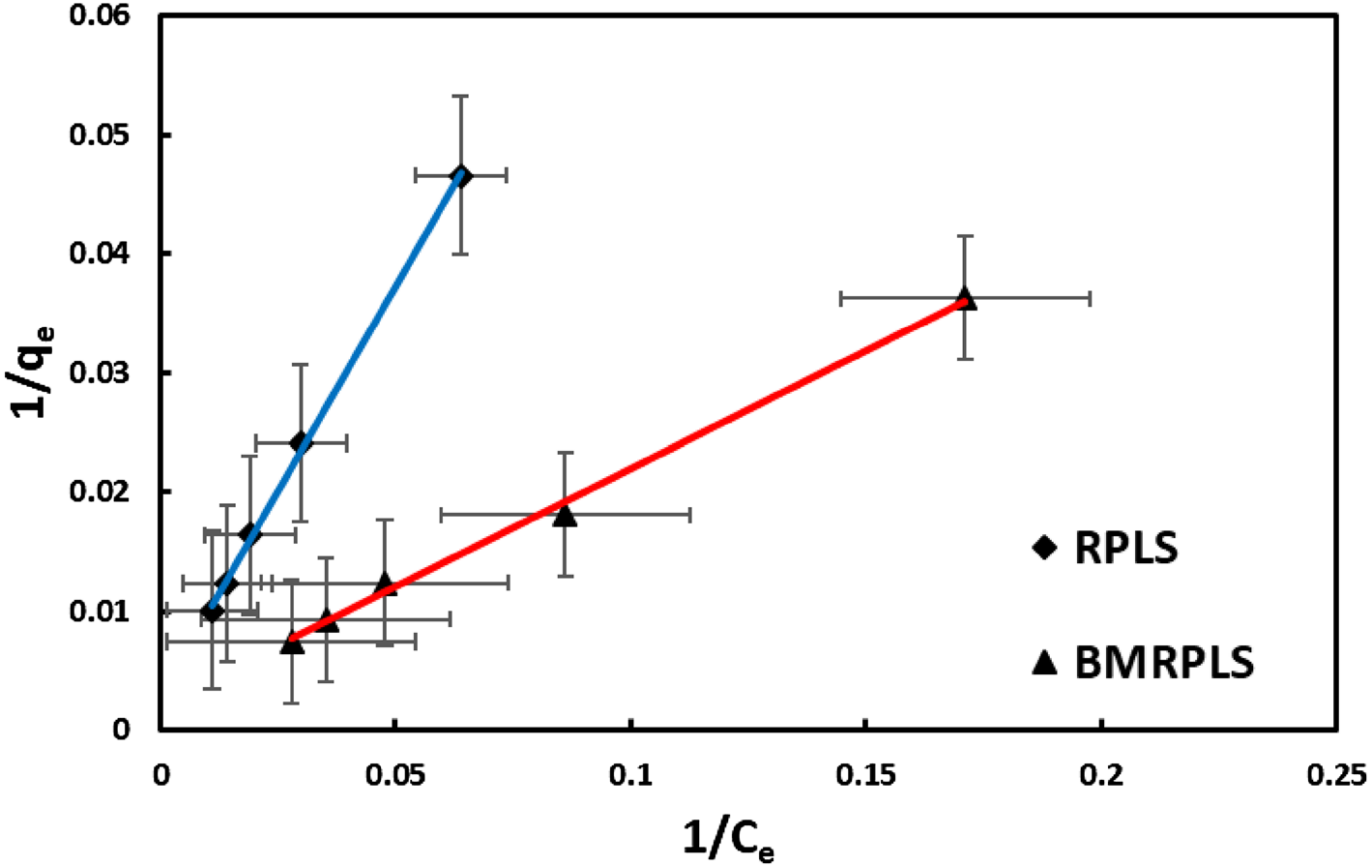
Langmuir adsorption isotherm.

The formation of a homogeneous monolayer of dye molecules on the adsorbent surface initiates the reactivity of the adsorbate, according to the Langmuir adsorption isotherm. Furthermore, it is stated that adsorption occurred exclusively at specific active locations and that no associations with adsorbed molecules and phase transitions occur^34^. Additionally, the adsorbed molecules cannot move across the surface or communicate with neighboring molecules. This kind of isotherm is indicated by the separation factor, R_L_ value, which also denotes the nature of adsorption. R_L_ values between 0 and 1 imply a favorable adsorption condition^35^.The maximal monolayer coverage capacity (q_max_) for the Langmuir isotherm model with standard deviation (Fig. 10) was calculated to be 344.83 mg/g and 454.55 mg/g for RPLS and BMRPLS. Table 3 represents the maximum adsorption capacity of different adsorption systems.

**Table 3:** Maximum adsorption capacity of different adsorption systems.

Adsorbent	Maximum adsorption capacity (mg/g)	Reference
Xanthated rice husk	90.02	^36^
Polyacrylamide‑grafted kiwi fruit peel powder	69.93	^37^
Fe_3_O_4_ decorated bacteria	114.8	^38^
White clove stem powder	1.95	^39^
NaOH-activated *Aerva javanica* leaf	315.20	^40^
Banana stem biochar	208.33	^41^
Red seaweed powder	52.6	^42^
*Moringa oleifera* seeds husk	469.55	^43^
RPLS	344.83	Present work
BMRPLS	454.55	Present work

Langmuir isotherm constant, K_L_ values for BMRPLS and RPLS correspond to 0.011173 and 0.004216 indicating that equilibrium sorption for both of 100 ppm dye solutions was favorable as R_L_ is 0.412955 for RPLS and 0.320789 for BMRPLS (R_L_ < 1). The adsorption data showed excellent agreement with the Langmuir isotherm model as shown by the R^2^ values of 0.9989 for RPLS and 0.9964 for BMRPLS.

#### Freundlich adsorption isotherm

**Figure 11:**
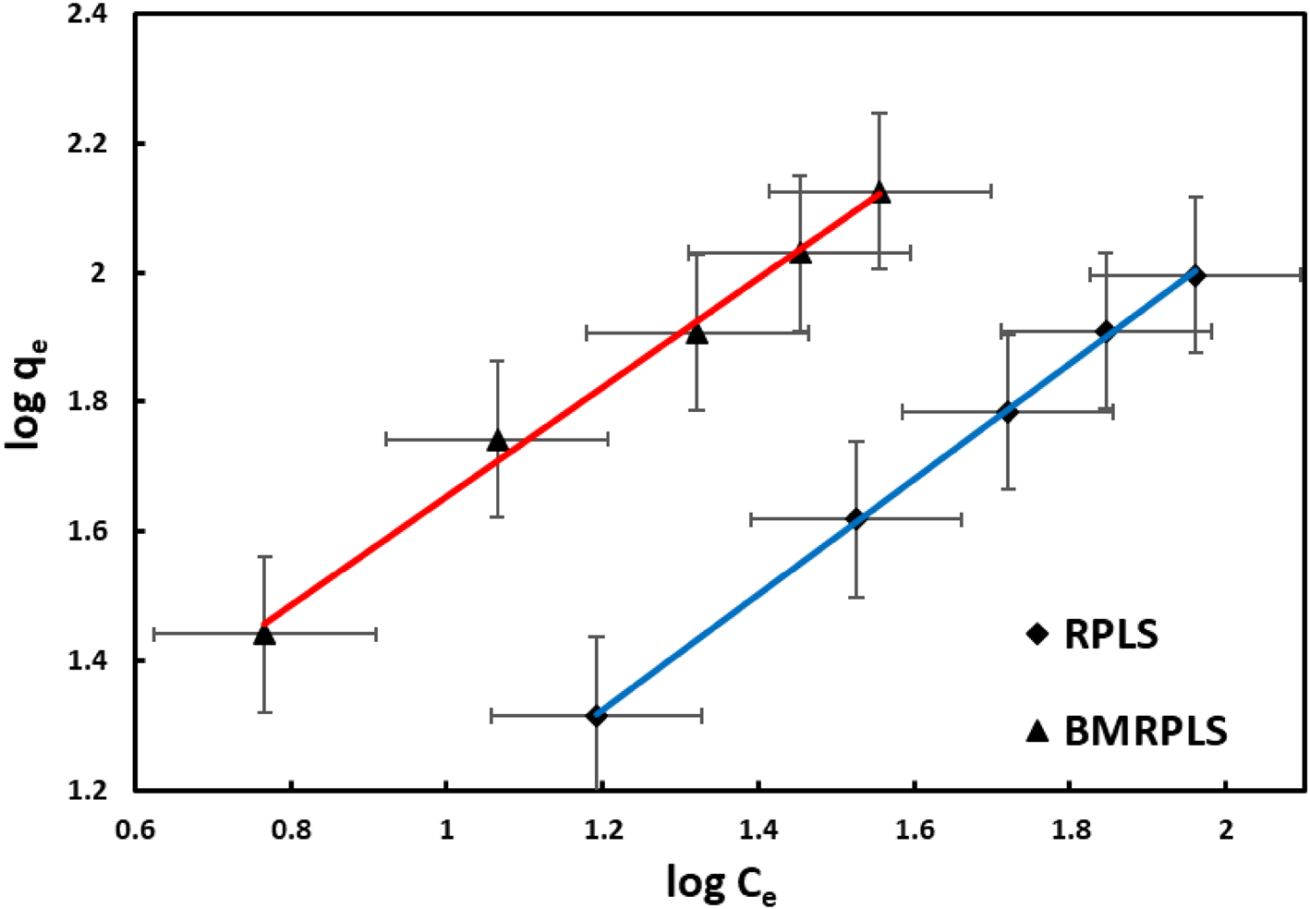
Freundlich adsorption isotherm.

The Freundlich isotherm has a closer link to the sorption equation that was empirically determined in 1912. The Freundlich model is frequently adopted when the adsorbent surface is heterogeneous. Figure 11 depicts the Freundlich plots with standard deviation of the royal palm/CV adsorption isotherms.

The Freundlich isotherm model was used to calculate the affinity of the CV adsorbate towards the leaf sheath adsorbent, K_F_. This affinity was calculated for BMPLS to be 2.24768 (L/mg), the adsorption strength or surface heterogeneity (1/n) 0.8432, and the R^2^ value 0.9995, indicating that the sorption results fit the model well. In addition, the Freundlich model predicts the affinity of the adsorbate towards the RPLS adsorbent, K_F_ to be 1.2896 (L/mg) as well as the surface heterogeneity (1/n), and the R^2^ value are all calculated suggesting that the sorption findings fit the model sound and comparable to each other. Therefore, the separation system (royal palm/CV) might be homogeneous/heterogeneous monolayer/multilayer adsorption results in higher adsorption capacity.

#### Temkin adsorption isotherm

**Figure 12:**
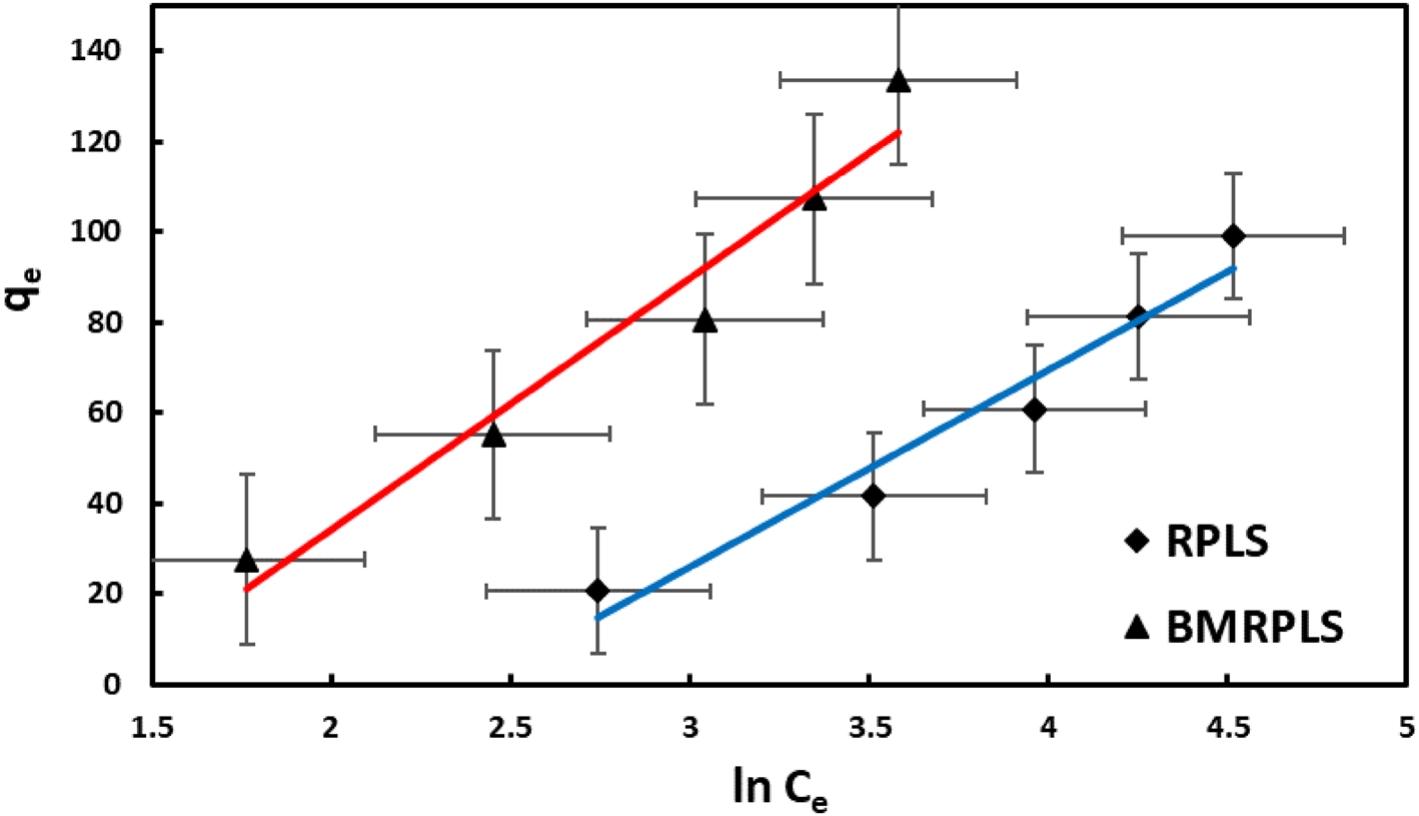
Temkin adsorption isotherm.

The Temkin adsorption isotherm shows that the adsorbate is adsorbed on energetic and non-equivalent adsorption sites on the surface of the adsorbent, with the more energetic sites serving as the initial sites of adsorption^44^. Figure 12 depicts the Temkin adsorption isotherm with standard deviation for the CV/RPLS systems.

This study determined the Temkin constant for BMRPLS to be 44.6265 (J/mol), which is connected to the heat of adsorption (b) from the Temkin isotherm model. The sorption data somehow fit the Temkin model comparing others, as shown by the Temkin isotherm equilibrium binding constant A (0.2512 L/g), B (55.518 J/mol), and R^2^ value (0.9534). This study determined the Temkin constant for RPLS to be 56.8146 J/mol, which is connected to the heat of adsorption b (J/mol) from the model. The sorption data of RPLS also fit the Temkin isotherm model along with binding constant A, B, and R^2^ value correspond to 0.0901 L/g, 43.608 J/mol and 0.9533. Table 4 shows the results of adsorption isotherm parameters of various models for RPLS and BMRPLS.

**Table 4:** Results of adsorption isotherm parameters of various models for RPLS and BMRPLS.

Isotherm	Parameter	RPLS	BMRPLS
Langmuir	q_max_ (mg/g)	344.8276	454.5455
	R_L_	0.4130	0.3208
	K_L_ (L/mg)	0.0042	0.0112
	R^2^	0.9989	0.9964
Freundlich	K_F_ (mg/g)	1.2896	2.2477
	n	1.1217	1.1860
	R^2^	0.9995	0.9939
Temkin	A (L/g)	0.0901	0.2512
	b (J/mol)	56.8146	44.6265
	B (J/mol)	43.608	55.518
	R^2^	0.9533	0.9534

The R^2^ value closer to unity indicates the better fit to the isotherm model^45^. According to R^2^ value it can be concluded that RPLS follows Freundlich adsorption model and BMRPLS trails Langmuir model better.

### Kinetic study

#### Pseudo–first order kinetics

**Figure 13:**
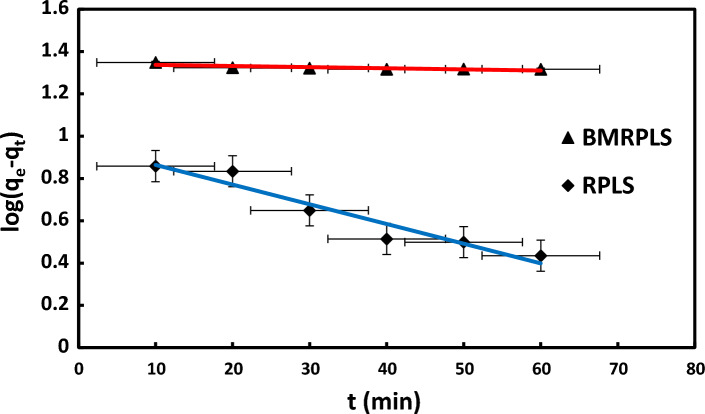
Pseudo-first order kinetics.

Figure 13 demonstrates the pseudo-first order kinetic model with standard deviation for the royal sheath and CV system. The values of ${k}_{1}$ and ${q}_{e}$ were calculated from the slopes and intercepts of the graphs of ${\text{log}}({q}_{e}-{q}_{t})$ vs. t as presented in the Figure. These extracted values with the correlation coefficients (${R}^{2}$) are listed in Table 4 point out that the estimated ${q}_{e}$ values are not in good agreement with that of the experimental values for both BMRPLS and RPLS adsorbents. However, the adsorption of CV on BMRPLS shows better fitting as well as follows the pseudo first order kinetic model well.

#### Pseudo–second order kinetics

**Figure 14:**
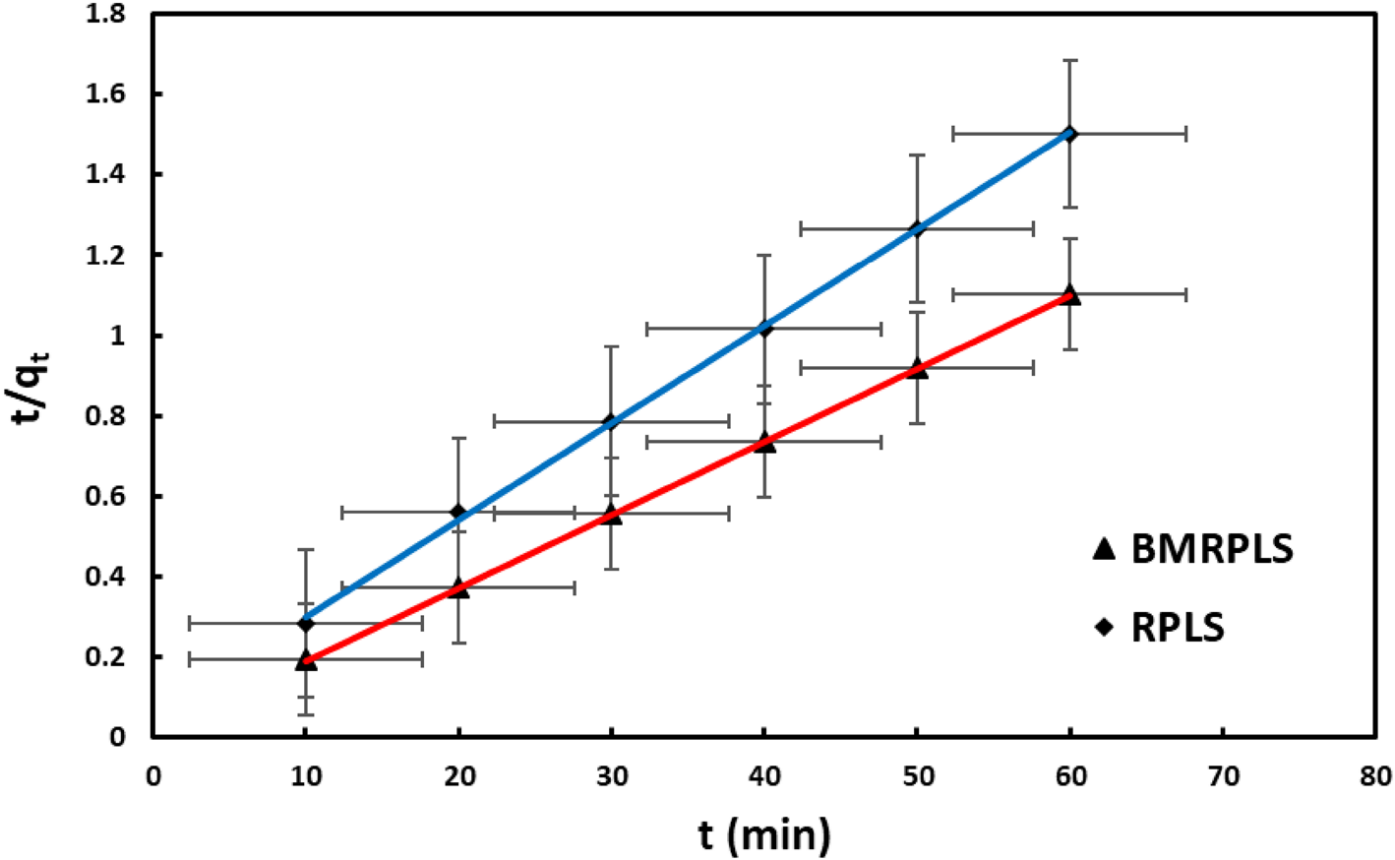
Pseudo-second order kinetics.

Plotting t/q_t_ vs. t with standard deviation yielded linear graphs, from which the slopes and intercepts of the plot at different concentrations were used to estimate q_e_ and ${k}_{2}$ (Fig. 14). The ${R}^{2}$ values for both the RPLS and BMRPLS obtained from the linear fitting to be 0.999 indicating the adsorption data fitted well with the kinetic equation as well as excellent agreement with the pseudo-second order model. In fact, adsorption rate is a function of adsorption capacity rather than adsorbate concentration^46^. Table 5 demonstrates the results of adsorption kinetic parameters of various models for RPLS and BMRPLS.

**Table 5:** Results of adsorption kinetic parameters of various models for RPLS and BMRPLS.

Model	Parameter	RPLS	BMRPLS
First order kinetics	K_1_ (min^–1^)	0.0093	0.0005
	R^2^	0.931	0.9864
Second order kinetics	q_e_ (mg/g)	42.6656	54.77
	K_2_ (min^–1^)	0.0098	0.037456
	R^2^	0.9993	0.9999
Intraparticle diffusion	K_in_ (mg/g.min^1/2^)	1.1056	5.0001
	C	31.479	51.034
	R^2^	0.9214	0.7493

#### Intraparticle diffusion model

**Figure 15:**
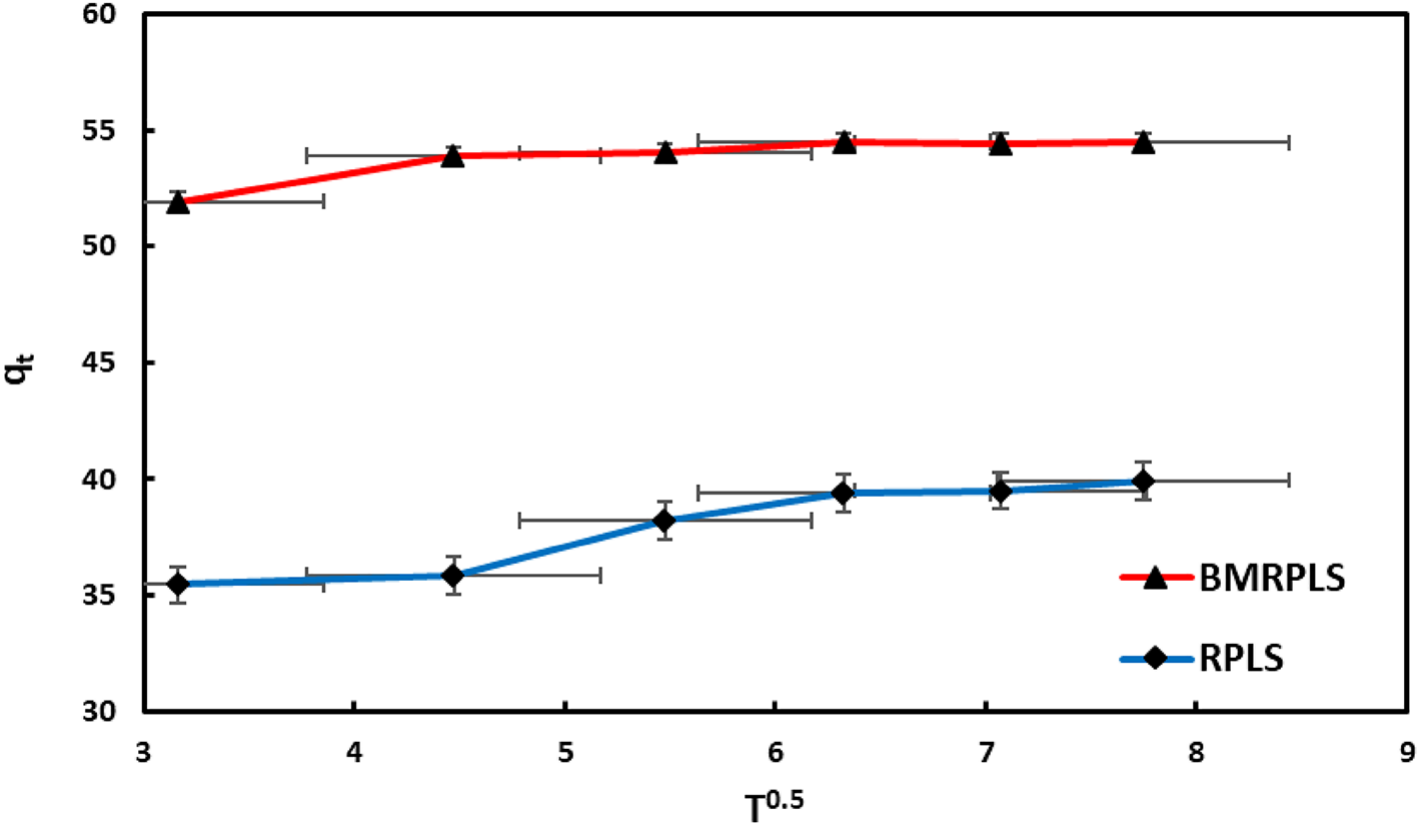
Intraparticle diffusion model.

The RPLS result demonstrates that the q_t_ vs. t^0.5^ plot with standard deviation (Fig. 15) can be split into three sections pointing out that in stage I, the adsorption capacity enriched quickly due to more accessible sites on the surface. The rate of CV adsorption in stage II was lower than in stage I. The external surface available locations were saturated, which explains the motive of the process. The adsorption reached stage III, where the diffusion model is not the rate-limiting phase^47^. The BMRPLS outcome demonstrates that the plot must be divided into two sections because it is not linear enough to span the entire time. The CV adsorption plot multilinearity demonstrates the multistage adsorption of CV on the BMRPLS. In this investigation, it is evident that neither the adsorption plot passes through the origin nor a primary rate-controlling step because it is not involved in the adsorption process. This outcome demonstrates that multiphases follow the adsorption process^48^. The rate constant of intraparticle diffusion was identified using the equation slope (K_in_). According to the K_in_ comparison, RPLS showed a considerably lower K_in_, indicating a slower rate of CV intraparticle diffusion. CV had higher intraparticle diffusion rates, as shown by the higher K_in_ for BMRPLS. When the adsorption process of BMRPLS achieved equilibrium, there was a high amount of intraparticle diffusion due to the high intraparticle diffusion rate and lengthy duration. Therefore, the greater degree of intraparticle diffusion of CV can be responsible for the higher adsorption capacity of BMRPLS than RPLS at high concentrations, as demonstrated elsewhere^49^. The surface sorption can be achieved in the rate-controlling phase having higher contribution when the C values are greater as reported in Ref.^23^. In the current study, BMRPLS has greater C value than RPLS which means BMRPLS surface sorption has greater contribution in adsorption rate controlling step. Table 5 shows the diffusion parameters of the model for RPLS and BMRPLS.

### Adsorption thermodynamics

**Figure 16:**
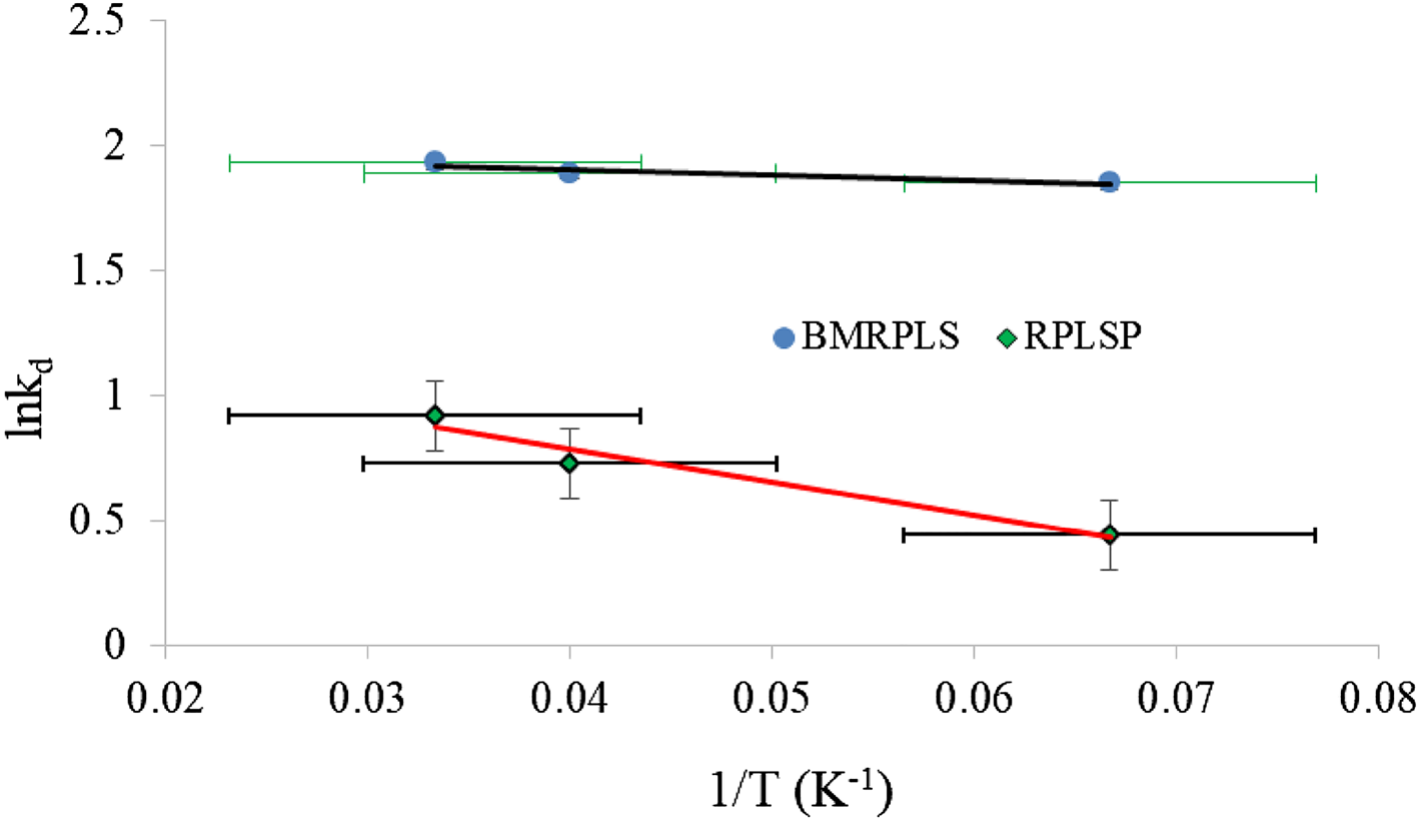
Plot of lnK_d_ versus 1/T for 100 ppm initial concentration.

Thermodynamic parameters are one of the most appropriate conditions for the adsorption process need to be developed considering the phenomenon that adsorbate molecules absorb into a porous solid surface with constant void fraction aimed at uniform distribution. The parameters considered in the current study are change in enthalpy (∆H°), entropy (∆S°) and Gibbs free energy (∆G°)^50^. ∆H° and ∆S° values were estimated from the slope and intercept of plot between lnK_d_ vs. 1/T with standard deviation for 100 ppm CV concentration (Fig. 16). The values of ∆H°, ∆S° and ∆G° are inserted in Table 6. The negative value of ∆H° indicates the exothermic nature of adsorption interaction between CV on to the RPLS and BMRPLS. The positive value of ∆S° shows the affinity of RPLS and BMRPLS for CV as well as enrichment in randomness at the adsorbent-CV interface. The negative ∆G° value indicates the feasibility and spontaneous nature of the process with a high preference of CV on to RPLS and BMRPLS^51^. BMRPLS has higher ∆H° than that of RPLS. The ∆S° of BMRPLS is also higher than RPLS and both have positive ∆S° values. The molecular affinity of the adsorbent to CV and randomness at the solid–solution interface is more pronounced for BMRPLS than RPLS during the adsorption process. The ∆G° values are negative for both the adsorbents. With lower ∆G° value of BMRPLS than that of RPLS shows CV adsorption on to the BMRPLS is more feasible process and spontaneous in nature.

**Table 6:** Thermodynamic parameters for adsorption of CV on RPLS and BMRPLS at initial concentration 100 ppm.

Adsorbent	∆H° (J/mol)	∆S° (J/mol.K)	∆G° (kJ/mol)		
			288 K	298 K	303 K
RPLS	− 110.776	10.943	− 1.052	− 1.801	− 2.311
BMRPLS	− 17.842	1.987	− 4.422	− 4.672	− 4.855

### Regeneration study

**Figure 17:**
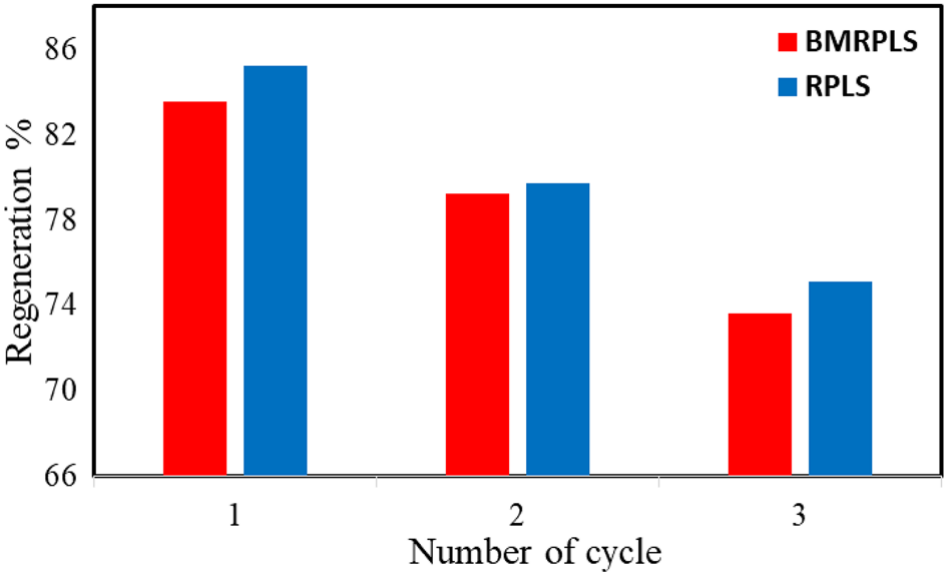
Regeneration of adsorbents RPLS and BMRPLS.

As shown in Fig. 17, the regeneration efficiency decreased with increasing number of cycles for both RPLS and BMRPLS. The regeneration efficiencies of RPLS and BMRPLS by distilled water after the 3rd cycle were approximately 73.61% and 75.12%, respectively. These results suggested that the regeneration of RPLS has better regeneration efficiency than BMRPLS in distilled water. The surface interaction parameters are particle size dependent. Ball milling causes reduction in particle size as well as possibly oxidized during milling in oxygen environment. Smaller particle size have a higher saturated surface concentration and a slower response to desorption^52^. This can be the fact behind the better regeneration efficiency of RPLS over BMRPLS.

## Conclusion

BMRPLS has been demonstrated in the manuscript to be an attractive adsorbent to remove cationic dyes from industrial wastewater even at low concentration and short time. Adsorption of CV dye onto RPLS and BMRPLS was studied and compared them quite effectively. CV adsorption of around 88% and 67% corresponding to BMRPLS and RPLS were observed at pH 6 and 60 min contact time. Various physicochemical parameters were determined and the sorption data fitted with Langmuir, Freundlich, and Temkin isotherms out of which BMRPLS fitted best in Langmuir model and RPLS showed best fitting for Freundlich model. The maximum adsorption capacity of BMRPLS and RPLS was 454.5 mg/g and 344.8 mg/g, respectively. Intraparticle diffusion model results in drop adsorption boundary layer of BMRPLS by the effect of ball milling. It can be concluded that BMRPLS is more active than RPLS being a model adsorbent headed for basic dyes towards practical application. 

## Data Availability

The datasets used and/or analysed during the current study are available from the corresponding author on reasonable request.
